# Cardioprotective Effect of Linalool against Isoproterenol-Induced Myocardial Infarction

**DOI:** 10.3390/life11020120

**Published:** 2021-02-05

**Authors:** Maged E. Mohamed, Mohamed S. Abduldaium, Nancy S. Younis

**Affiliations:** 1 Department of Pharmaceutical Sciences, College of Clinical Pharmacy, King Faisal University, Al-Ahsa 31982, Saudi Arabia; nyounis@kfu.edu.sa; 2 Department of Pharmacognosy, College of Pharmacy, Zagazig University, Zagazig 44519, Egypt; 3 Department of Cardiology, College of Medicine, Zagazig University, Zagazig 44519, Egypt; drmohsafwat81@gmail.com; 4 Department of Pharmacology, Zagazig University, Zagazig 44519, Egypt

**Keywords:** apoptosis, essential oils, inflammatory markers, ischemic heart disease, monoterpene alcohols

## Abstract

Background: Myocardial infarction (MI), a life-threatening disorder, arises from the imbalance between oxygen supply and myocardial demand. Linalool is a naturally occurring monoterpenes with proved numerous pharmacological actions. This study investigated the cardioprotective effect of Linalool on isoproterenol (ISO)-induced MI in rat models and explored part of the underlying molecular mechanisms. Methods: Rats were divided into five groups; groups I and II served as normal and linalool control groups, Group III administered ISO alone; groups V and VI received two different doses of Linalool and were challenged by ISO. Different biochemical parameters were determined, including hemodynamic, infarction size, cardiac enzymes, apoptotic markers, and inflammatory mediators. Results: Linalool limited the infarcted area size and diminished the elevated cardiac enzymes. Linalool escalated HO-1 and Nrf2, both nuclear and cytosol fractions, and reduced Keap 1. Linalool enhanced cardiac antioxidant activities, reduced inflammatory cytokines (tumor necrosis factor-alpha (TNF-α), nuclear factor-κ-B (NF-κB), interleukin 1 beta (IL-1β), interleukin 6 (IL-6)), apoptotic markers (Caspase-3, Caspase-9, and Bax), and elevated Bcl2. Conclusion: Linalool could act as an effective cardioprotective agent in the MI model through improving the oxidative condition, probably via the Nrf2/HO-1 pathway and by abolishing both apoptotic and inflammatory responses.

## 1. Introduction

Ischemic heart diseases (IHDs) are among the most threatening diseases to human life due to high rates of attack and associated disability and mortality. Myocardial infarction (MI), a common presentation of IHD, is an acute disorder arising from an imbalance between oxygen supply and myocardium demand [1]. Isoproterenol (ISO) administration in experimental animals provided a rapid, simple, and non-invasive method to generate myocardial damage status similar to that seen in humans with acute MI. In addition, ISO produced a model that had low mortality, high reproducibility, and validity compared with other animal models, which make it more appropriate for the assessment of potential cardioprotective agents [2]. The main mechanism involved in isoproterenol-induction of myocardial ischemia is the generation of free radicals, reactive oxygen species, lipid peroxidation, oxidative stress, and calcium overload, which lead to the alteration in membrane permeability, causing apoptosis and necrosis and finally slowing the conduction between myocardial cells, triggering alterations in heart electrical activity [2,3].

Nrf2 is a redox-sensitive transcription factor that binds to the antioxidant response elements (AREs) to promote cell survival gene Bcl-2 expression, prevent apoptosis, regulate mitochondrial function, and stimulate antioxidant enzymes expression to reduce oxidative damage [3]. Under oxidative stress or other potentially damaging stimuli, Nrf2 dissociates from Keap1 in the cytoplasm and translocates into the nucleus, leading to the transcriptional activation of phase II enzymes/antioxidant genes, including HO-1, glutathione S transferase (GST), and glutamate cysteine ligase (GCL), etc. [1]. Li et al. [4] demonstrated that the pharmacological activation of Nrf2 significantly reduced the oxidative stress in cardiomyocytes. Furthermore, the effects of cardioprotective agents, such as 4-hydroxy-2-nonenal and MG132 (a proteasome inhibitor), were abolished in Nrf2-knockout mice, together with the expression of the downstream target of Nrf2 signaling pathway [1].

Diverse essential oils and other biological substances extracted from natural resources have been targets for new drug discovery and have established a wide variety of biological actions [5]. Linalool is one of the most abundant naturally occurring monoterpenes, located in the essential oil fraction of numerous plants of aromatic species [6]. Linalool has been distinguished by the Food and Drug Administration as a safe product to be used for flavoring and as an adjuvant for human consumption [7]. However, linalool can be toxic when ingested in large doses (more than 500 mg/kg/day in rodents) [8]. Linalool has been evidenced to have abundant pharmacological actions, including antidepressant [6], antifungal [9], gastro-protective [10], anti-nociceptive [11], and anti-tumor [12] activities. In addition, linalool protects against smoking-induced lung inflammation through inhibiting NF-κB activation [13] and, similarly, inhibits Lipo-polyssaccharide (LPS)-induced inflammation in BV2 microglia cells [14]. Linalool has been proven to retain a protective effect in several models of neurodegeneration, such as in the triple transgenic mice model of Alzheimer disease (AD) (3xTg-AD) [15], the global cerebral ischemia model [16], and glutamate and N-methyl-D-Aspartate (NMDA) oxicity [17]. Furthermore, linalool reverses the histopathological hallmarks of AD and restores cognitive and emotional functions via an anti-inflammatory effect [15].

The main purpose of the current study is to explore the cardioprotective effect of the monoterpene alcohol, linalool, on MI through administrating the drug in ISO-induced MI rat models. The existing investigation is assumed to reveal some of the molecular mechanisms underlying this cardio-protective effect of linalool by evaluating the expressions and activities of cardiac, inflammatory, apoptotic, and antioxidant markers.

## 2. Materials and Methods

### 2.1. The Essential Oil Materials

The essential oil of lavender (*Lavandula angustifolia,* family Lamiaceae) was purchased from commercial sources in Alahsaa, Eastern province, Kingdom of Saudi Arabia, in May 2020. The commercial oil was extracted by shaking with diethyl ether (HPLC Grade, Merck) at the ratio of 1 oil to 3 diethyl ether parts. The extraction was done three times, and all the diethyl ether was pulled together and evaporated under reduced pressure (25 °C) to give the pure lavender oil (yield 82%).

### 2.2. Lavender Essential Oil Analysis

The isolated and purified lavender essential oil was subjected to GC-MS analysis to validate identity. Gas chromatography/flame ionization (GC/FID) analysis was performed using GC-2010 Plus (Shimadzu Corporation, Kyoto, Japan) gas chromatograph, equipped with FID-2010 Plus detector and a split/splitless injector. The column was RTX-5MS^®^fused silica capillary (30 m × 0.25 mm i.d and 0.25 μm film thickness); the oven temperature initially was held at 45 °C for 2 min and then increased to 280 °C at 4 °C/min. The carrier gas was helium with a flow rate of 2 mL/min; the temperature of the injector and detector were 250 °C and 300 °C, respectively. The split ratio was 1:20 and the injection volume was 5 μL. Quantification of essential oil components was performed by relative parentage area calculations (area normalization). The relative percentage of the essential oil constituents was assessed on the basis of the FID responses from the total peak area, using percentage area normalization [18,19,20].

Gas chromatography/mass spectrometry (GC/MS) data were recorded on GCMS-QP2010 Plus (Shimadzu corporation, Kyoto, Japan). The ionization energy for the mass spectrometer was 70 eV and the split ratio was 1:30. The other conditions were identical to those mentioned for GC/FID. Kovat’s retention indices (RI) were calculated with respect to a set of co-injected standard hydrocarbons (C10-C28, Sigma Aldrich, Steinheim, Germany). Compounds, including geraniol, were identified by comparing their spectral data and retention indices with Wiley Registry of Mass Spectral Data 10th edition (April 2013), NIST 11 Mass Spectral Library (NIST11/2011/EPA/NIH), and literature data [21]. Major components of the purified lavender oil are mentioned in Table 1.

### 2.3. Isolation of Linalool from the Purified Essential Oil of Lavender

Linalool was separated from the purified lavender essential oil using normal column chromatography techniques, according to Savira, et al. [22], with some amendments. Silica gel was used as a stationary phase, the sample to stationary phase ratio was 1:20, the sample weight was 10 gm, and the mobile phase was benzene: ethyl acetate (95: 5, *v*/*v*). The isocratic elution mode was used at a flow rate of 3 drops per second, and eluent was collected in vials, each at 50 mL. The elution was monitored by thin-layer chromatography (TLC) (Silica gel 254 nm, same mobile phase as the column), and visualization was done using ultraviolet (UV) light at 254 nm and by using anisaldehyde/sulphuric acid reagent or iodine vapor.

### 2.4. Animals

Male Wister rats (weight: 230–250 g) were purchased from the animal house facility, King Saud University, Riyadh. The animals were kept at standard laboratory conditions with a free access to a pellet diet and tap water ad libitum throughout the whole experiment.

### 2.5. Ethical Statement

All animal investigational protocols and practices were applied according to the Ethical Conduct for Use of Animals in Research Guidelines in King Faisal University and following Animal Research Ethics Committee permission at King Faisal University (KFU-REC/2019-3-20).

### 2.6. Experimental Design

Animals were randomly distributed into five groups (*n* = 6). Groups I and II represented normal and linalool control groups, in which animals were administered either saline or linalool for 30 days and then saline (s.c) on the last 2 days of the experiment (28th and 29th days). Group III represented myocardial infarction (MI) control (ISO), in which animals were given saline orally for 30 days then administered isoproterenol (ISO, Cat. No I6504, Sigma Aldrich) (85 mg/kg, s.c.) on the 28th and 29th days to induce MI. Groups IV and V represented linalool pretreatment, in which the animals were given linalool (either 50 or 100 mg/kg) [5] orally for 30 days and then provoked with ISO (85 mg/kg s.c.), as in group III (ISO + Linalool).

### 2.7. Detection of Hemodynamic Parameters

Following 24 h from the last dose of ISO, animals were anesthetized using urethane in a dose of 1.5 g/kg [23]. Animals were placed in a noninvasive computerized electrocardiogram (ECG) apparatus (Kent Scientific, Torrington, CT, USA) to acquire ECG recordings. ECG constituents, including the ST segment, P wave QT, P-R and R-R intervals, and QRS complex, were electronically measured and obtained. Blood pressure (BP) was then measured, using a noninvasive onscreen tail-cuff system (Emka Technologies’ Systems, France), as mentioned previously [23], to obtain systolic arterial pressure (SAP), diastolic arterial pressure (DAP), mean arterial pressure (MAP), and heart rate (HR).

### 2.8. Sample Collection

Subsequent to ECG and BP measurements, animals were sacrificed, blood samples were collected, and hearts were dissected, weighted, and stored. The acquired blood samples were centrifuged (15 min/6000 rpm/4 °C) to separate the serum from different experimental groups, which was then frozen at −80 °C for further biochemical investigations.

### 2.9. Infarct Size Determination

Hearts separated from different experimental groups were cut into 4–5 transverse cuts, incubated in 10% triphenyl tetrazolium chloride (TTC) (dissolved phosphate buffer, pH 7.4, 30 min, room temperature, dark room), and then fixed with 10% formalin [1]. Infarcted areas were quantified using Image J^®^ program (National Institutes of Health, University of Wisconsin).

### 2.10. Biochemical Parameters Assessment

Samples from the obtained heart tissues were homogenized in 10% phosphate buffer to measure numerous biochemical parameters. ELISA kits were consumed to measure cardiac enzymes; creatine phosphokinase (CPK; ab187396), creatine phosphokinase heart (CK-MB; ab187396), cardiac troponin T (cTnT; ab246529), and cardiac troponin I (cTnI; ab246529); apoptotic markers; caspase 3 (ab39401), Caspase 9 (ab65608), and inflammatory mediators; tumor necrosis factor-alpha (TNF-α; ab46070), interleukin 6 (IL-6; ab100772), interleukin 1 beta (IL-1β; ab100768), and nuclear factor-κ-B (NFκB; ab176648) (all obtained from Abcam, Eugene, OR, USA), following the manufacturer’s protocols and using a microplate reader (SpectraMax i3x, Molecular Devices, USA).

### 2.11. Western Blot Analysis

Frozen hearts were homogenized in RIPA buffer, containing protease inhibitor, which was centrifuged (10,000 rpm, 10 min, 4 °C), and then the protein content in the gained supernatant was measured by NanoDropLitespectrophotometer (Thermo Fisher Scientific, Waltham, MA, USA). Protein samples (45 µg) were electrophoresed in SDS-PAGE, transported to the polyvinylidene fluoride (PDVF) membrane, incubated with 5% bovine serum albumin (BSA) first and then with primary antibodies of HO-1, nuclear Nrf2, cytosolic Nrf2, Keap1, and β actin (1:1000). These primary antibodies were identified using horse-radish peroxidase (HRP)-conjugated secondary antibodies. Antigen-antibody reaction was pictured with an enhanced chemiluminescence (ECL; Sigma Aldrich, St. Louis, MO, USA) kit under a gel documentation system. Image J software was used to evaluate the acquired images.

### 2.12. Real-Time PCR

Real-time PCR was preformed according to the technique described elsewhere [23]. Briefly, the Trizol reagent kit (Invitrogen, Waltham, MA, USA) and reverse transcription polymerase chain reaction (RT-PCR) kit were used to purify total RNA and inverse transcription reaction, respectively. In total 20 μL of the reaction volume was mixed with 1 μL total RNA (1 μg/µL), incubated at 42 °C for 15 min, followed by 95 °C for 2 min, and the generated cDNA was stored at −20 °C. In total, 50 μL of PCR reaction mixture enclosed × 50 ROX Reference Dye (1 μL), sense and antisense primers (1 μL each, primers are mentioned below), ×2 SYBR Green PCR Master Mix (25 μL), cDNA template (4 μL), and sterilized distilled H2O (18 μL). The PCR reaction condition incorporated pre-denaturing at 95 °C for 10 s, then 40 cycles of 95 °C/5 s, and 60 °C/30 s and 72 °C/1 min. Quantification analyses were completed via Opticon-2 Real-time PCR reactor (MJ Research, Reno, NV, USA). Step PE Applied Biosystems (Perkin Elmer, Waltham, MA, USA) software was used to analyze real time-PCR results. Expression of the target gene was measured and correlated to the reference gene (β-actin).

### 2.13. Statistical Analysis

Data are presented as mean ± SD. An unpaired *t*-test was used to compare two different treatment groups, while multiple comparisons were performed using one-way ANOVA, followed by Tukey Kramer as a post hoc test, as appropriate. The 0.05 level of probability was used as the criterion for significance. All statistical analyses were performed using GraphPad Prism (GraphPad Prism Inc., La Jolla, CA, USA) software, version 5.

## 3. Results

### 3.1. Isolation of Linalool from Lavender Volatile Oil

**Figure 1:**
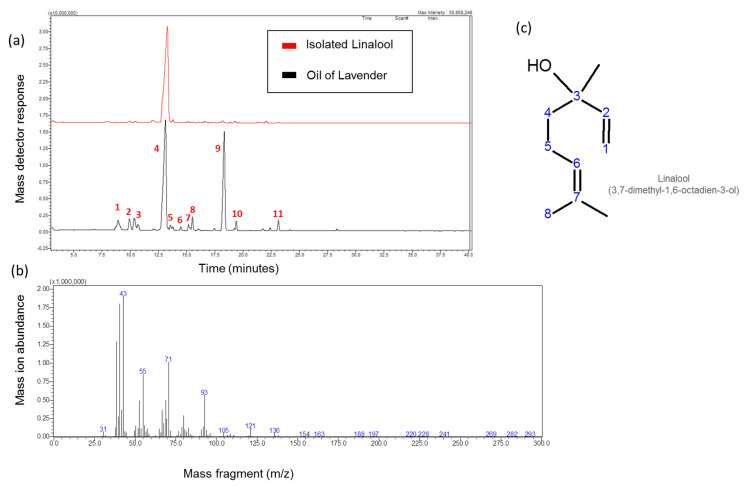
Isolation of linalool from lavender volatile oil. (**a**) GC-MS chromatogram of lavender (*L. angustifolia*) volatile oil and isolated linalool. (**b**) Mass spectrogram of the isolated linalool. (**c**) Chemical structure of linalool. Numbers on (**a**) are related to compounds in Table 2.

The commercial oil of lavender was purified using diethyl ether to extract the oil components away from adulterants (if any), and the percentage yield of the purified oil reached 82% of the original commercial oil volume. Linalool represented 47.24% of the purified lavender volatile oil, an elevated percentage that was enough to encourage its isolation via column chromatography procedures (Figure 1a). Referring to Table 2, the percentage of linalyl acetate, an acetate ester of linalool, in the purified oil was 29.15%, which inspired the idea to increase the content of linalool through hydrolysis of linalyl acetate. In total, 1 gm of the purified oil was refluxed in 2% aqueous HCl for 2 h, and the reflux components were extracted three times with diethyl ether and subjected to GC-MS analysis (data not shown). The data indicated no significant increase in the linalool contents, the Linalyl acetate signal nearly disappeared, and the appearance of a new peak (Rt = 16.285 min, RI = 1188) assigned for α-terpineol. Referring back to literature, it was reported that the reflux of linalyl acetate in acidic artificial gastric juice was rapidly hydrolyzed (t1/2 < 5 min) to linalool, which rapidly rearranges into α-terpineol [7].

The purified lavender volatile oil was subjected to column chromatography, as mentioned in the methodology section. Fractions (20–27) of the eluent produced a major spot on TLC (Rf 0.31). These fractions were pulled together, and the excess solvent was evaporated under reduced pressure to produce an oily, colorless solution with characteristic odor. The isolated compound was identified by GC-MS using the retention index (Rt = 13.14 min, RI = 1098, Table 2, Figure 1a) and a library search with a molecular weight of m/z 154.25 (Figure 1b), with a library identity percentage of 91% of 3, 7-Dimethylocta-1,6-dien-3-ol (linalool), Figure 1c. The purity of the compound was checked using GC-MS and TLC, and finally, the pure compound was subjected to FT-IR, ^1^HNMR, and ^13^C NMR for more detailed structure elucidation.

### 3.2. Effect of Linalool on Infarcted Area and Heart to Body Weights Ratio

**Figure 2:**
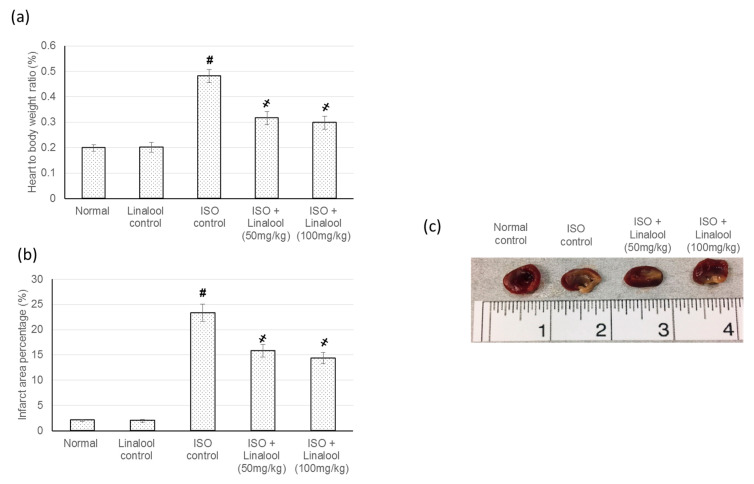
Effect of pretreatment with linalool (50 and 100 mg/kg) for 30 days in ISO-induced myocardial infarcted animal actions on (**a**) heart to body ratio, (**b**) infarction size percentage, and (**c**) illustrative images of heart slices from different experimental groups stained with triphenyl tetrazolium chloride (TTC). All values were stated as mean ± SD (*n* = 6). The probability value is *p* < 0.05, where # indicates statistical significance from the normal control group, ҂ indicates statistical significance from the isoproterenol (ISO)-induced myocardial infarction (MI group), using one-way ANOVA, followed by Tukey’s test as a post-hoc analysis.

Animals in the control groups exhibited negligible infarcted areas, while animals in the ISO group revealed a major infarcted area. On the other hand, pretreatment with linalool (50 or 100 mg/kg) showed a significant infarcted area limiting effect, reaching 32.2% and 38.5% decreases compared to the infarcted area of ISO animals, Figure 2. Furthermore, linalool (50 or 100 mg/kg) showed a substantial decline in the increased heart weight to body weight ratio that occurred in rats suffering from induced MI. However, no significant difference was detected between the two doses of linalool in either the infarcted area percent change or the heart to body weights ratio, as shown in Figure 2.

### 3.3. Effect of Linalool on Hemodynamic Parameters

**Figure 3:**
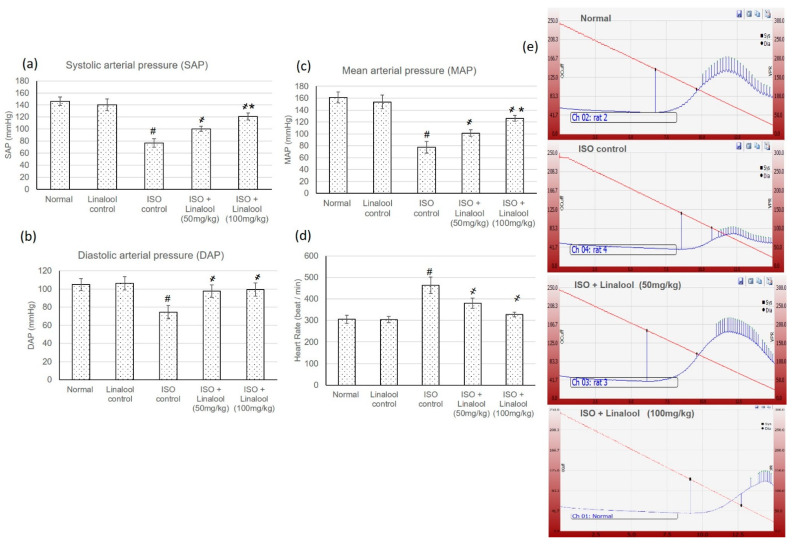
Effect of pretreatment with linalool (50 and 100 mg/kg) for 30 days in ISO-induced myocardial infarcted animal actions on (**a**) systolic arterial pressure (SAP), (**b**) diastolic arterial pressure (DAP), (**c**) mean arterial pressure (MAP), and (**d**) heart rate (HR). (**e**) Demonstrative pictures of blood pressure from different experimental groups. All values were stated as mean ± SD (*n* = 6). The probability value is *p* < 0.05, where # indicates a statistical significance from the normal control group, ҂ indicates a statistical significance from the ISO-induced MI group, and * indicates a statistical significance from the linalool (50 mg/kg) group using one-way ANOVA, followed by Tukey’s test as a post-hoc analysis.

**Figure 4:**
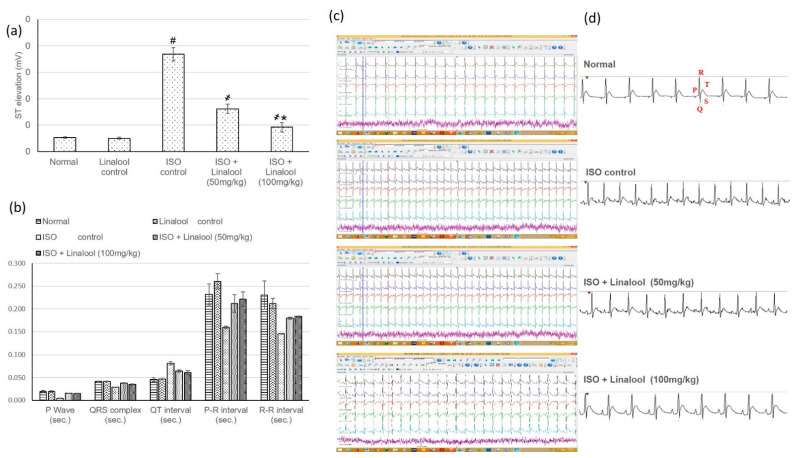
Effect of pretreatment with linalool (50 and 100 mg/kg) for 30 days in ISO-induced myocardial infarcted animal actions on (**a**) ST elevation, (**b**) electrocardiogram (ECG) constituents, including P wave, QRS complex, QT interval, and P-R and R-R intervals. (**c**,**d**) ECG demonstrative images. All values were stated as mean ± SD (*n* = 6). The probability value is *p* < 0.05, where # indicates statistical significance from the normal control group, ҂ indicates statistical significance from the ISO-induced MI group, and * indicates statistical significance from the linalool (50 mg/kg) group, using one-way ANOVA, followed by Tukey’s test as a post-hoc analysis.

Normal and linalool control groups showed normal heart rate (HR), blood pressure (BP), and electrocardiographic (ECG) patterns, whereas the ISO group displayed an extensive HR intensification and BP indices reduction, together with plentiful ECG alterations, including a ST segment and QT interval widening, and shortening in the P wave, QRS complex, and P-R and R-R intervals, compared to normal animals, as shown in Figure 3 and Figure 4, respectively. Pretreatment with both doses of linalool (50, 100 mg/kg) reduced HR and amended ECG and BP alterations.

### 3.4. Effect of Linalool on Cardiac Indicators

**Figure 5:**
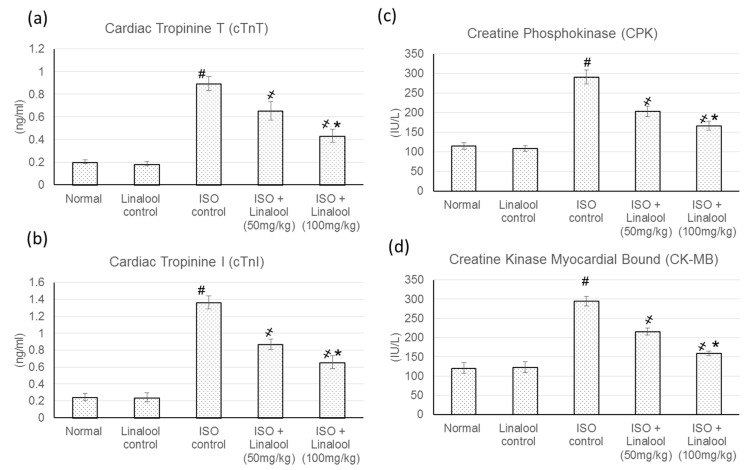
Effect of pretreatment with linalool (50 and 100 mg/kg) for 30 days in ISO-induced myocardial infarcted animal actions on (**a**) cardiac troponin T (cTnT), (**b**) cardiac tropinine I (cTnI), (**c**) creatine phosphokinase (CPK), and (**d**) creatine kinase-myocardial bound (CK-MB). All values were stated as mean ± SD (*n* = 6). The probability value is *p* < 0.05, where # indicates statistical significance from the normal control group, ҂ indicates statistical significance from the ISO-induced MI group, and * indicates statistical significance from the linalool (50 mg/kg) group, using one-way ANOVA, followed by Tukey’s test as a post-hoc analysis.

ISO animals showed a substantial intensification in the cardiac indicator enzymes compared to the control groups. Conversely, pretreatment with linalool (50, 100 mg/kg) significantly diminished these elevations in cardiac enzymes, with a percentage reduction reaching 27.03% and 51.45% for cTnT, 36.20% and 52% for cTnI, 30.5% and 43% for CPK, and finally, 26.85% and 46.07% for CK-MB, as represented in Figure 5.

### 3.5. Effect of Linalool on Keap1/Nrf2/HO-1 Pathway

**Figure 6:**
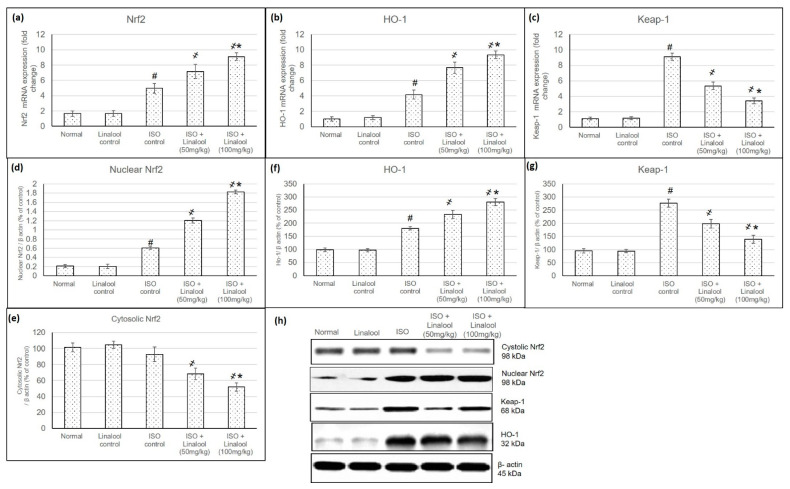
Effect of pretreatment with linalool (50 and 100 mg/kg) for 30 days in ISO-induced myocardial infarcted animal actions on mRNA expression of Nrf2 (**a**) HO-1 (**b**), and Keap1 (**c**), and protein expression of nuclear Nrf2 (**d**), cytosolic Nrf2 (**e**), HO-1 (**f**), and Keap-1 (**g**). The western blot images for all the mentioned parameters are shown in (**h**). Lower section: Photos of protein-gel electrophoresis. All values were stated as mean ± SD. The probability value is *p* < 0.05, where # indicates statistical significance from the normal control group, ҂ indicates statistical significance from the ISO-induced MI group, and * indicates statistical significance from the linalool (50 mg/kg) group, using one-way ANOVA, followed by Tukey’s test as a post-hoc analysis.

The mRNA and protein expression levels of Keap1, cytosolic Nrf2, nuclear Nrf2, and HO-1 were assessed to evaluate linalool’s ability to activate a Nrf2 antioxidant signaling pathway in myocardial tissues. Results are shown in Figure 6. The mRNA and protein expression of Keap1 was significantly elevated in the myocardial tissues of ISO-induced MI, while the altered expressions were normalized in linalool pretreatment groups (in (Figure 6a,d). A challenge with ISO non-significantly reduced cytosol Nrf2 protein expression level and this alteration was further significantly promoted in linalool pretreated groups. On the other hand, ISO-induced MI caused a substantial increase in nuclear accumulation of Nrf2, whereas linalool pretreatment significantly resulted in a further increase in accumulation of Nrf2 in the nuclear fraction as compared with ISO animals (Figure 6b–f). mRNA and protein expression of HO-1 were increased in ISO-induced MI animals and were further intensified with linalool pretreatment (Figure 6c,g).

### 3.6. Effect of Linalool on Antioxidant Activities

**Figure 7:**
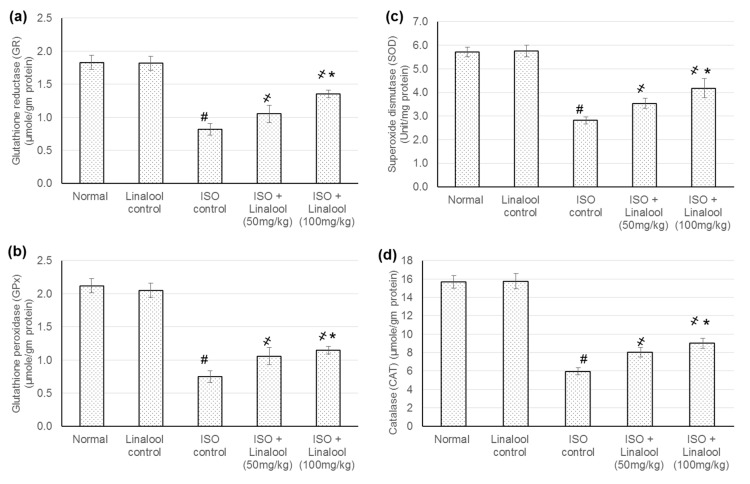
Effect of pretreatment with linalool (50 and 100 mg/kg) for 30 days in ISO-induced myocardial infarcted animal actions on (**a**) glutathione reductase (GR), (**b**) glutathione peroxidase (GPx), (**c**) superoxide dismutase (SOD), and (**d**) catalase (CAT). All values were stated as mean ± SD. The probability value is *p* < 0.05, where # indicates statistical significance from the normal control group, ҂ indicates statistical significance from the ISO-induced MI group, and * indicates statistical significance from the linalool (50 mg/kg) group, using one-way ANOVA, followed by Tukey’s test as a post-hoc analysis.

In ISO-induced MI animals, cardiac antioxidants enzyme levels, such as glutathione reductase (GR), glutathione peroxidase (GPX), superoxide dismutase (SOD), and catalase (CAT) were significantly lessened, while animals supplemented with the linalool (50 and 100 mg/kg) for 30 days prior to MI demonstrated a marked enhancement (*p* < 0.05) in the activities of cardiac antioxidants, reaching a percentage elevation of 16.6% and 43.75% for GR, 41.7% and 53.59% for GPX, 25.68 and 48.52% for SOD, and finally, 35.12% and 51.54% for CAT, as shown in Figure 7.

### 3.7. Effect of Linalool on Inflammatory Indicators

**Figure 8:**
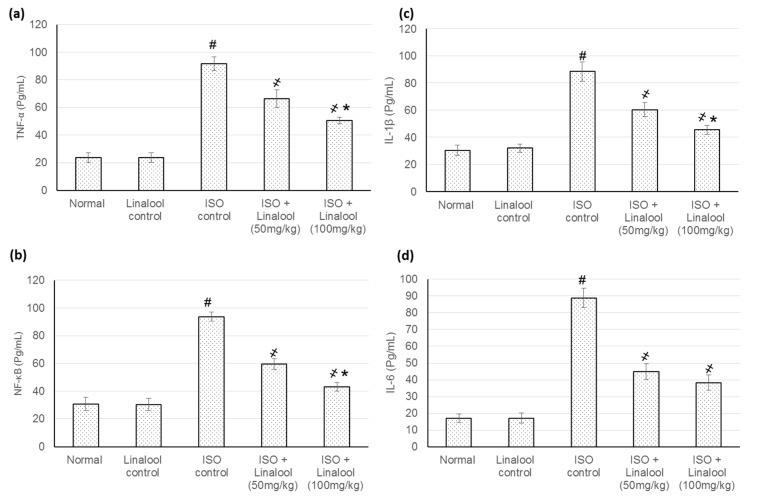
Effect of pretreatment with linalool (50 and 100 mg/kg) for 30 days in ISO-induced myocardial infarcted animal actions on (**a**) tumor necrosis factor-alpha (TNF-α), (**b**) nuclear factor-κ-B (NF-κB), (**c**) interleukin 1 beta (IL-1β), and (**d**) interleukin 6 (IL-6). All values were stated as mean ± SD. The probability value is *p* < 0.05, where # indicates statistical significance from the normal control group, ҂ indicates statistical significance from the ISO-induced MI group, and * indicates statistical significance from the linalool (50 mg/kg) group, using one-way ANOVA, followed by Tukey’s test as a post-hoc analysis.

As displayed in Figure 8, numerous inflammatory cytokines, such as TNF-α, NF-κB, IL-1β, and IL-6, within the cardiac homogenate, were enlarged extensively (*p* < 0.05) as a result of ISO-induced MI. In total, 30 days of management with linalool (50 and 100 mg/kg), earlier to ISO administration, markedly dropped all the enzyme levels (*p* < 0.05) and produced a percentage reduction of 30% and 40.6% for TNF-α, 33% and 54.06% for NF-κB, 30.84% and 43.09% for IL-1β, and, finally, 49.22% and 53.23% for IL-6 levels.

### 3.8. Effect of Linalool on the Apoptotic Markers

**Figure 9:**
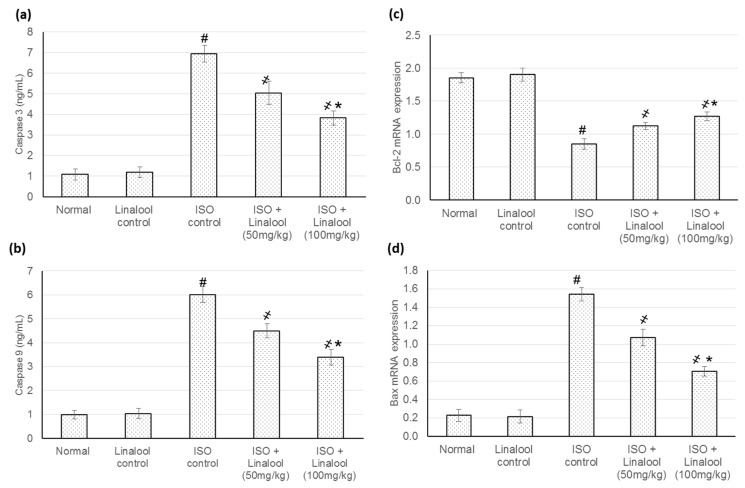
Effect of pretreatment with linalool (50 and 100 mg/kg) for 30 days in ISO-induced myocardial infarcted animal actions on (**a**) Caspase-3, (**b**): Caspase-9, (**c**) Bcl2, and (**d**) Bax. All values were stated as mean ± SD. The probability value is *p* < 0.05, where # indicates statistical significance from the normal control group, ҂ indicates statistical significance from the ISO-induced MI group, and * indicates statistical significance from the linalool (50 mg/kg) group, using one-way ANOVA, followed by Tukey’s test as a post-hoc analysis.

Figure 9 indicates linalool actions on the myocytes apoptotic indicators in different investigational groups. An exponential boost in Caspase-3, Caspase-9, and Bax mRNA expression levels, as well as a reduced Bcl2 mRNA expression level, were recognized in animals experienced with MI, caused by ISO administration. Nevertheless, linalool (50 and 100 mg/kg) resulted in a decrease in Caspase-3 Caspase-9, and Bax, respectively, when compared to ISO-induced MI animals. In addition, Bcl2 level was increased with linalool (50 and 100 mg/kg) administration, respectively, when compared to the ISO-induced MI group.

## 4. Discussion

The improved understanding of the pathophysiological and biochemical processes involved in MI has stimulated the exploration for new preventive or therapeutic agents that could efficiently limit and/or treat myocardial damage.

In the current study, animals experiencing myocardial infarction (MI) revealed a major infarcted area and cardiac enzymes intensification, which resulted in numerous cardiac function alterations, as established by HR intensification and BP indices reduction, together with plentiful ECG modifications. On the other hand, linalool illustrated an infarcted area-limiting effect and diminished the elevated cardiac enzymes with subsequent improvement in cardiac function, as demonstrated from HR, ECG, and BP amendments.

Linalool reduced blood pressure, probably due to a direct action on the vascular smooth muscle, leading to vasodilation [24]. Previous reports have demonstrated that linalool has a direct action on the vasculature and an inducing effect on endothelium-dependent vasorelaxation in mouse thoracic aortae through activating soluble guanylyl cyclase and K+ channels [25]. In addition, linalool attenuates a hypertension progress, decreased cardiac hypertrophy, improved vasodilatory function, and decreased vasoconstriction in spontaneously hypertensive rats (SHR), confirming linalool as a potential antihypertensive compound [5].

One of the proposed mechanisms explaining the underlying biochemical and molecular stages during MI is the nuclear factor erythroid 2-related factor 2 (Nrf2), which has been demonstrated to be a critical transcription factor that regulates the induction of phase 2 detoxifying and antioxidant genes occurring with ISO administration [1]. Consequently, the next step, in this study, was to estimate linalool’s ability to activate the Nrf2 antioxidant signaling pathway in myocardial tissues, through measuring mRNA and protein expression levels of Keap1, cytosolic Nrf2, nuclear Nrf2, and HO-1. mRNA and protein expression of Keap1, nuclear Nrf2, and HO-1 were significantly elevated, while cytosolic Nrf2 protein expression was non-significantly reduced in the myocardial tissues of ISO-induced MI animals, indicating that ISO caused Nrf2 activation due to the presence of ROS, which by itself has the ability to activate Nrf2. A substantial rise in Keap1 expression was observed in ISO-induced rats, signifying that Nrf2 was more associated with Keap1 with less translocation into the nucleus to activate antioxidant gene expression. Earlier studies showed that mitochondria-derived Reactive oxygen species (ROS) induced the activation of Nrf2 and Keap1 and overexpression of Nrf2 abolished reactive oxidants, whereas reduced Nrf2 activity exhibited the opposite effects [1,4]. Linalool consumption prior to the MI insult succeeded to significantly escalate HO-1 and Nrf2 both nuclear and cytosolic levels, while Keap 1 was reduced as compared with ISO-induced rats. Earlier studies proved that linalool activated the Nrf2/HO-1 signaling pathway, which resulted in the inhibition of LPS-induced inflammatory mediators in BV2 microglia cells [14].

Cardiac tissue levels for antioxidant markers, such as GR, GPX, SOD, and CAT, were significantly lessened in animals challenged with ISO, while those pre-supplemented with linalool showed elevated antioxidant marker quantities. This escalation in antioxidant marker concentrations could be attributed to the accumulation of Nrf2 in the nucleus, which promoted the transcription of key antioxidant markers and phase II enzymes and contributed to cellular protection against oxidative stress and to the potentiated antioxidant defense capacity in cells. However, this elevation in the antioxidant marker level could also be ascribed to a direct stimulation of linalool on the transcription of such markers. Previous studies have shown that linalool prevents lipid peroxidation and loss of antioxidants depletion in several models, such as in UVB-exposed human skin cells [26], glutamate, and NMDA-induced neurotoxicity toxicity [17].

ISO has resulted in the elaboration of several inflammatory cytokines, such as TNF-α, NF-κB, IL-1β, and IL-6, within the cardiac tissue homogenate, while the management with linalool markedly dropped the levels of such cytokines. Several earlier investigations reported the anti-inflammatory actions of linalool. For example, linalool diminished cigarette smoke (CS)-induced lung inflammation, suppressed the infiltration of inflammatory cells and TNF-α, IL-6, IL-1β, IL-8, and Monocyte chemoattractant protein-1 (MCP-1) production, and inhibited CS-induced NF-κB activation [13]. In addition, linalool inhibited LPS-induced TNF-α, IL-1β, NO, and PGE2 production and NF-κB activation in BV2 microglia cells [14].

A major downstream regulation of the apoptotic death signal resides with the Bcl-2/Bax gene family [26]. The current study verified an exponential boost in Caspase-3, Caspase-9, and Bax, as well as a reduced Bcl2 mRNA expression level in ISO control animals. Nevertheless, linalool cause a reduction in Caspase-3, Caspase-9, and pro-apoptotic protein Bax, as well as an increase in anti-apoptotic protein, Bcl-2, when compared to ISO-induced MI animals. Similarly, linalool treatment prevented UVB-induced apoptotic signaling, probably through preventing DNA damage and subsequent apoptotic activation [26].

## 5. Conclusions

Linalool, the acyclic monoterpene alcohol, was scrutinized in this study as a cardioprotective agent against myocardial infarction insult. Linalool succeeded keeping the heart function after MI, as demonstrated in hemodynamic parameters and infarction area analysis. Linalool mostly protected the myocardial tissues and cell from the ischemic effects by adjusting the oxidative status via the Nrf2/HO-1 pathway and, at the same time, managing both inflammatory and apoptotic responses of the cardiac cells. This study could suggest using linalool, either purely or as a component of edible herbs as a protective therapy for patients with incidence to myocardial infarction.

## Figures and Tables

**Table 1:** Primer sequences used in this study were as follows.

Markers	Primer Sequence (5′ to 3′)	
	Forward Primer	Reverse Primers
Bcl-2	5′-CCGGGAGATCGTGATGAAGT-3′	5′-ATCCCAGCCTCCGTTATCCT-3′
Bax	5′-GTGGTTGCCCTCTTCTACTTTG-3′	5′-CACAAAGATGGTCACTGTCTGC-3′
Nrf2	5′-CATTTGTAGATGACCATGAGTCGC-3′	5′-ATCAGGGGTGGTGAAGACTG-3′
Keap1	5′-CTTCGGGGAGGAGGAGTTCT-3′	5′-:CGTTCAGATCATCGCGGCTG-3′
HO-1	5′-GTGCACATCCGTGCAGAGAA-3	5′-GTGCACATCCGTGCAGAGAA-3′
β-actin	5′-TGACAGGATGCAGAAGGAGA-3′	5′-TAGAGCCACCAATCCACACA-3′

**Table 2:** Major compounds in the purified lavender oil and their area percentages.

No.	Rt (min)	RI	Area %	Compound Name	Molecular Formula
1	8.94	995	4.39	3-Octanone	C_8_H_16_O
2	9.955	1035	3.40	Limonene	C_10_H_16_
3	10.715	1037	1.52	(Z)-β-Ocimene	C_10_H_16_
4	13.14	1098	47.24	(R)-Linalool	C_10_H_18_O
5	13.595	2003	1.67	α-Terpinolene	C_10_H_16_
6	14.5	1150	0.70	(+/-)Camphor	C_10_H_16_O
7	15.19	1155	1.68	Isoborneol	C_10_H_18_O
8	15.555	1176	2.38	Terpinen-4-ol	C_10_H_18_O
9	18.345	1258	29.15	Linalyl acetate	C_12_H_20_O_2_
10	19.415	1381	2.10	Geranyl acetate	C_12_H_20_O_2_
11	23.155	1410	1.92	Trans-Caryophyllene	C_15_H_24_

## Data Availability

All data created or used during this study are openly available in this manuscript.
